# Comparison of Janani Suraksha Yojana (JSY) and augmented Arogya Laxmi scheme (ALS) in improving maternal and child health outcomes in urban settlements of Hyderabad, South India

**DOI:** 10.1186/s12884-024-06381-7

**Published:** 2024-03-08

**Authors:** E R Nandeep, Raja Sriswan Mamidi, Sreenu Pagidoju, Spandana Pamidi, Mahesh Kumar Mummadi, Venkata Raji Reddy G, Chinta Khadar Babu, Samarasimha Reddy N, JJ Babu Geddam

**Affiliations:** 1 Clinical Epidemiology Division, Indian Council of Medical Research-National Institute of Nutrition (ICMR-NIN), Hyderabad, India; 2 Maternal and Child Health, Nutrition Division, Indian Council of Medical Research-National Institute of Nutrition (ICMR-NIN), Hyderabad, India; 3 Model Rural Health Research Unit, Chandragiri, Tirupathi India

**Keywords:** Janani Suraksha Yojana, Arogya Laxmi Scheme, India, Maternal and child health, Deliveries, Conditional cash transfers

## Abstract

**Background:**

India accounts for the largest number of global neonatal deaths with around 20 per 1000 live births. To improve the utilization of government services for institutional deliveries, Augmented Arogya Laxmi Scheme (ALS) was launched in Telangana state of southern India. This study assessed the effectiveness of the Janani Suraksha Yojana (JSY), which combines cash assistance with delivery and post-delivery care, in comparison to ALS in improving the outcomes related to antenatal, natal, and postnatal care in urban settlements of Hyderabad, Telangana, southern India.

**Methods:**

This was a two-year cross-sectional study conducted in 14 urban settlements of Hyderabad city from September 2017- August 2019. All mothers delivered during the 18 months preceding the survey were enrolled after a written informed consent. Field investigators collected data on variables related to socio-demographic characteristics, awareness, and utilization of JSY and ALS programs. Variables related to antenatal history, antenatal care, complications during birth, delivery outcomes, newborn care, and postnatal care till 28 days were assessed. We used multivariable logistic regression model to examine the association between the different maternal, child, and socio-demographic characteristics of the two study groups.

**Results:**

A total of 926 mothers were beneficiaries of Janani Suraksha Yojana (JSY) program while 933 mothers were beneficiaries of augmented Arogya Laxmi Scheme (ALS). Mothers in ALS group (AOR 1.71; 95% CI 1.21–2.43) were at increased odds of having more than eight antenatal care (ANC) visits compared to the mothers availing JSY. Mothers in ALS group were at decreased odds of having complications like severe pain in the abdomen (AOR 0.43; 95% CI 0.22–0.86), swelling of legs or feet (AOR 0.59; 95% CI 0.44–0.80) compared to mothers in JSY group. Children of mothers in the ALS group had increased odds of receiving breastfeeding within 30 minutes of birth (AOR 1.46; 95% CI 1.13–1.88) compared to children of mothers in JSY group.

**Conclusions:**

The newly launched augmented ALS led to the increased utilization of the government health facilities and improved the maternal and child health outcomes.

## Background

According to the National Family Health Survey-5 data from India (NFHS-5), the Neonatal Mortality Rate (NMR) is 16.8 per 1000 live births [1]. India accounts for the largest number of global neonatal deaths at 20 per 1000 live births [2]. Almost 40% of neonatal deaths are happening at the time of labour and the first 24 h after delivery with the most common cause being pre-maturity (35%) [3]. As per the national sample registration system (SRS), Maternal Mortality Rate in India is 113 deaths per 100,000 live births (2016-18) [4] while the Sustainable Development Goals-Target 3.1 is to reduce the global maternal mortality ratio to under 70 per 100,000 live births by the year 2030 [5].

Institutional deliveries with appropriate hygienic conditions and life-saving devices can help reduce morbidity and mortality of both mother and child [6]. 97% of all the deliveries in Telangana were institutional, out of which only 49.7% were in public health facilities [1]. Accessing private health facilities for childbirth brings high out-of-pocket-expenditures (OOPE) [7]. Conditional Cash Transfer (CCT) is often seen as an effective way to improve the rates of Institutional deliveries, and thereby pregnancy outcomes in developing countries [8]. The Indian government initiated Janani Suraksha Yojana (JSY scheme) to promote institutional deliveries to reduce neonatal and maternal mortality and includes cash incentive with delivery and post-delivery care [9]. Even though the government is providing both health care and monetary benefits, early registrations (< 12 weeks of gestation) of pregnant women and utilization of government services for institutional deliveries were poor in both urban and rural areas [10].

The government of Telangana launched the Augmented Arogya Laxmi Scheme (ALS), which included components of the Maternal and Child Health (MCH) kit, for pregnant and lactating mothers on 2nd June 2017 with an objective of reducing both NMR and maternal death rate by providing a monetary benefit of Rs.12,000/- for mothers delivering a baby boy and Rs.13,000/- for mothers delivering a baby girl child. A newborn care kit is provided immediately after delivery [11]. This monetary benefit which is given in three instalments is in addition to the Rs. 6000/- they receive from JSY(including the monetary benefit from PMMVY: Pradhan Mantri Matru Vandana Yojana [12]). State procures JSY money and include them in the MCH Kit. The first instalment worth Rs. 4000/- is given upon successful completion of early ANC registration (< 12 weeks) and 5 ANC visits. The second instalment, which is given at the time of delivery in the government facility includes Rs.4000/- (baby boy) or Rs.5000/- (baby girl) along with newborn care kit which contains 12 different items useful for the child and the mother. The third instalment of Rs. 4000/- is given upon successful completion of child’s immunization and this monitory benefit will be monitored at the Anganwadi Centre (AWC) [11, 13, 14].

According to the United Nation World Urbanization prospects 2018, the urbanization in India is 34% in 2018, and is projected to be about 36% in 2022 and cross the 50% mark in 2046 [15, 16]. Formation of slums is one the first visible effects of urbanization. It results in cities not able to provide migrants with areas to live that have basic amenities [17]. Urban health, especially of the urban economically weaker sections have received less focus compared to the rural health in India [18]. The health status of the urban slum dwellers are poor, and the access of reproductive and child health services are far from adequate [19]. Slums in urban areas tend to have health indicators that are below the average in other urban areas [19]. With this background, the current study was conducted with the objective to compare the effectiveness of Arogya Laxmi scheme in comparison to the beneficiaries of the JSY program in improving the indicators related to antenatal, natal, and postnatal care in urban settlements of Hyderabad.

## Methods

### Study setting and design

This was a two-year cross-sectional study in 14 urban settlements of Hyderabad city from September 2017- August 2019. Hyderabad is the capital city of Telangana State, located in the south-central part of India. The city lies in the Deccan Plateau and has an average height of 536 m above the sea level. The city spans over 650 square kilometers, and has a population of more than six million [20]. Greater Hyderabad Municipal Corporation oversees the civic infrastructure of the city [20]. Hyderabad has 1466 slums with a population of more than 1.8 million according to the survey conducted by Greater Hyderabad Municipal Council in 2009 [20, 21]. Hyderabad district contains three area hospitals, 14 urban nutrition health clusters, and 85 upper primary health centers [22]. The settlements with higher percentage of home deliveries and less likelihood to migrate were selected and the settlements were evaluated as least, moderate, and extremely vulnerable based on the distance of the settlements from health center and health vulnerability. As augmented Arogya Laxmi Scheme (with MCH kit) was launched in June 2017, during the initial phases of the study, the enrolment had only beneficiaries under JSY scheme. Later during the second half of the data collection, we were able to enroll the beneficiaries of ALS. The study was done similar to a pre- and post-intervention study. Pre-intervention being only JSY, and post being the Augmented ALS. All the deliveries that happened before 2017 were beneficiaries of only JSY. During the initial phase, as the new program of Augmented ALS was only three months old, we excluded all new cases and included only those participants who gave birth prior to the launch of this program. This constituted the enrolment of the participants in JSY scheme. After enrolment in the JSY group, we recruited mothers who have been part of the new Augmented ALS. Therefore, we were able to compare two different groups based on the timeline of program delivery.

### Data collection

A pre validated and structured questionnaire containing socio-demographic characteristics, awareness and utilization of JSY and ALS programs, antenatal history, and antenatal care was used. The questionnaire was standardized after repeated discussions with the experts and was piloted before starting the study ensuring good quality of the collected data. Data were collected on the complications of mothers during the pregnancy, delivery, and newborn care. Complications during birth and post-partum till 28 days were also collected. Data were also collected on the immunization history of the child, history of any illness to the mother or child in the last 15 days, place of ANC visits, status of Tetanus Toxoid (TT) injections to the mothers, history of iron and folic acid supplementation: if availed and consumed, and place and type of delivery.

### Statistical analysis

The data were entered into the computer using Census and Survey Processing System (CS Pro 7.0.2). We have used CS Pro with conditional checks to handle missing values and outliers. Analysis was conducted using Statistical Package for Social Sciences (SPSS) 19.0 for windows. Using Boxplot and Z Score (normalized), we have identified outliers and considered them as missing values for minimum or maximum values for each variable. (Boxplot criteria: low outliers are below Q1 − 1.5 ⋅ IQR & high outliers are above Q3 + 1.5 ⋅ IQR; Z Score criteria: considered an outlier when the z-score exceeds + 3 or is less than − 3. The Z-score criteria of nutritional statuses are based on the WHO Anthro software. The data were normally, independently, and identically distributed for continuous variables.). The two groups of JSY and ALS were compared for all the baseline characteristics. Chi-square test was used to compare the characteristics of the two groups. Univariate analysis was performed to test the association between socio-demographic characteristics, maternal and child health outcomes between the two groups. All the variables with *p*-value less than 0.25 on univariate analysis and variables of clinical and contextual importance were used to build a multivariable logistic regression model. Backward elimination feature as well as background knowledge were used for variable selection. Potential multicollinearity was identified by reviewing the correlation matrix for the predictor variables. Correlation coefficient with an absolute value > 0.7 was typically considered a strong correlation between the predictor variables. For all analysis, *p* < 0.05 was considered statistically significant.

## Results

### Socio demographic characteristics of study population

926 mothers were the beneficiaries of JSY program and 933 mothers were the beneficiaries of Arogya Laxmi Scheme (ALS). In JSY group, 728 (79%) mothers and in ALS group 774 (83%) were Hindus. In JSY group, 444 (48%) mothers belong to Other Backward Class and in ALS group 514 (55%) mothers belong to Other Backward Class (Table 1).

**Table 1:** Socio- demographic characteristics of children and mothers participated in the survey among the urban settlements of Hyderabad (*N* = 1859)

Variable	Category	GROUP				*P* value*
		JSY (*N* = 926)		ALS (*N* = 933)		
		Number	(%)	Number	(%)	
Religion		*n* = 925		*n* = 931		
	Hindu	728	78.7	774	83.1	< 0.001
	Muslim	137	14.8	129	13.9	
	Christian/Others	60	6.5	28	3	
Social Class		*n* = 925		*n* = 931		
	Schedule Caste (SC)	246	26.6	223	24	< 0.001
	Schedule Tribe (ST)	123	13.3	164	17.6	
	Other Backward Class (OBC)	444	47.9	514	55.2	
	General/others	112	12.1	30	3.2	
Number of family members		*n* = 923		*n* = 931		
	1–2	449	48.6	490	52.6	0.017
	3–4	420	45.5	410	44	
	5+	54	5.9	31	3.3	
Family type		*n* = 925		*n* = 931		
	Nuclear	559	60.4	507	54.5	0.032
	Extended Nuclear	166	17.9	197	21.2	
	Joint	200	21.6	227	24.4	
Type of house		*n* = 925		*n* = 932		
	Kutcha	100	10.8	54	5.8	< 0.001
	Semi pucca	542	58.6	635	68.1	
	Pucca	186	20.1	197	21.1	
	Open Space/ squatter hut	97	10.5	46	4.9	
House ownership		*n* = 925		*n* = 932		
	Own	421	45.5	408	43.8	0.552
	Rented	379	41	405	43.5	
	Migrant camp/living in other house	125	13.5	119	12.8	
Number of rooms (excluding kitchen)		*n* = 925		*n* = 930		
	1	416	45	419	45.1	0.480
	2	308	33.3	328	35.3	
	3 and above	201	21.7	183	19.7	
Type of cooking fuel		*n* = 925		*n* = 932		
	Liquid Petroleum Gas (LPG)	811	87.7	886	95.1	< 0.001
	Wood/Coal/cow dung	91	9.8	39	4.2	
	Kerosene	23	2.5	7	0.8	
Source of Drinking water		*n* = 925		*n* = 932		
	Improved	449	48.5	514	55.2	0.004
	Unimproved	476	51.5	418	44.8	
Toilet facility		*n* = 925		*n* = 932		
	Improved	828	89.5	869	93.2	0.004
	Unimproved	97	10.5	63	6.8	
Wealth Index		*n* = 918		*n* = 924		
	Highest	231	25.2	255	27.6	0.491
	High	144	15.7	140	15.2	
	Middle	168	18.3	183	19.8	
	Low	230	25.1	220	23.8	
	Lowest	145	15.8	126	13.6	

*Chi square test was used **P < 0.05 considered statistically significant

### Maternal characteristics comparison between the two groups JSY and ALS

Majority of mothers in JSY group (58%) and ALS group (70%) were in 19–24 years age group followed by 25–30 years age group (JSY (37%) and ALS (28%)). There were differences in terms of complications during pregnancy such as severe pain in abdomen (2% in ALS vs. 6.6% in JSY), swelling of legs or feet during pregnancy being less in ALS group compared to JSY and were statistically significant. ALS group had 539 (57.8%) normal vaginal deliveries compared to 467 (50.5%) in JSY group. A statistically significant difference was also found in terms of place of delivery with 923 (99%) mothers in ALS group delivering at a government hospital compared to 517 (55.9%) mothers in JSY group (Table 2).

**Table 2:** Comparison of maternal characteristics, antenatal visits, Antenatal Complications, and delivery details between the beneficiaries of Janani Suraksha Yojana and Arogya Laxmi scheme in urban settlements of Hyderabad (*N* = 1859)

Variable	Category	GROUP				*P* value*
		JSY (*N* = 926)		ALS (*N* = 933)		
		N	(%)	N	(%)	
**Maternal characteristics**						
Mother’s Age (years)		*n* = 925		*n* = 932		
	≤ 18	13	1.4	8	0.9	< 0.001
	19–24	537	58.1	649	69.6	
	25–30	340	36.8	260	27.9	
	> 30	35	3.8	15	1.6	
Mother’s Education		*n* = 925		*n* = 932		
	Illiterate/No formal education	215	23.2	173	18.6	0.017
	Up to Primary/ 5–9 class	229	24.8	232	24.9	
	Secondary school	352	38.1	411	44.1	
	Graduate, Postgraduate & above	129	13.9	116	12.4	
Mother’s occupation		*n* = 925		*n* = 932		
	Employee	83	9	24	2.6	0.071
	Home maker/ Currently not working	842	91	908	97.4	
**Antenatal care**						
Place of Antenatal Care (ANC)		*n* = 907		*n* = 926		
	Sub-centre /Primary Health Centre (PHC)/Government Hospital	661	72.9	919	99.2	0.001
	Private Hospital	246	27.1	7	0.8	
Total number of ANC visits		*n* = 923		*n* = 929		
	≤ 8	194	21	90	9.7	< 0.001
	> 8	729	79	839	90.3	
TT doses		*n* = 919		*n* = 931		
	One dose	18	2	20	2.1	0.774
	Two doses	901	98	911	97.9	
Haemoglobin (g/ dl)		*n* = 792		*n* = 551		
	< 10	171	21.6	109	19.8	0.422
	≥ 10	621	78.4	442	80.2	
		*n* = 837		*n* = 915		
AWC services utilization	Yes	745	89	830	90.7	0.238
	No	92	11	85	9.3	
**Complications during pregnancy**						
High blood pressure during pregnancy		*n* = 925		*n* = 930		
	Yes	68	7.4	51	5.5	0.101
	No	857	92.6	879	94.5	
Severe pain in the abdomen		*n* = 925		*n* = 932		
	Yes	61	6.6	19	2	< 0.001
	No	864	93.4	913	98	
Breathlessness during pregnancy		*n* = 925		*n* = 932		
	Yes	54	5.8	43	4.6	0.236
	No	871	94.2	889	95.4	
Swelling of legs or feet during pregnancy		*n* = 925		*n* = 932		
	Yes	241	26.1	155	16.6	< 0.001
	No	684	73.9	777	83.4	
**Delivery Related Variables**						
Gestational age at delivery (in weeks)		*n* = 924		*n* = 924		
	< 37	154	16.7	178	19.3	0.146
	≥ 37	770	83.3	746	80.7	
Type of delivery		*n* = 925		*n* = 932		
	Normal	467	50.5	539	57.8	< 0.001
	Caesarean	458	49.5	393	42.2	
Place of delivery		*n* = 925		*n* = 932		
	Home	36	3.9	6	0.6	< 0.001
	Government hospital	517	55.9	923	99	
	Private Hospital	372	40.2	3	0.3	
IFA tablets supplementation during lactation		*n* = 922		*n* = 930		
	Yes	840	91.1	760	81.7	< 0.001
	No	82	8.9	170	18.3	

*Chi square test was used **P < 0.05 considered statistically significant

### Comparison of child characteristics between the two groups

In both the groups, most of the children were in the age group of 7–12 months. In ALS group 592 (63.7%) children were breastfed within 30 min of the birth compared to 513 (55.7%) in JSY group and this difference was statistically significant. In ALS group 901 (97%) children were given colostrum compared to 857 (93.1%) children in JSY group. Burden of stunting was low in ALS group (20.7%) compared to JSY group (38.6%) (Table 3).

**Table 3:** Comparison of Neonatal and infant characteristics between the beneficiaries of Janani Suraksha Yojana and Arogya Laxmi scheme in urban settlements of Hyderabad (*N* = 1859)

Variable	Category	GROUP				*P* value*
		JSY (*N* = 926)		ALS (*N* = 933)		
		N	(%)	N	(%)	
Gender of the Child		*n* = 925		*n* = 932		
	Male	496	53.6	499	53.5	0.972
	Female	429	46.4	433	46.5	
Current age of child in months		*n* = 923		*n* = 929		
	≤ 6	26	2.8	312	33.6	< 0.001
	7–12	467	50.6	419	45.1	
	> 12	430	46.6	198	21.3	
Birth Weight (in grams)		*n* = 919		*n* = 932		
	< 2500	109	11.9	97	10.4	0.320
	≥ 2500	810	88.1	835	89.6	
Birth Order		*n* = 921		*n* = 932		
	1	339	36.8	373	40	< 0.001
	2	367	39.8	493	52.9	
	3	156	16.9	52	5.6	
	4 and above	59	6.4	14	1.5	
Breast feeding within 30 min of birth		*n* = 921		*n* = 930		
	Yes	513	55.7	592	63.7	< 0.001
	No	408	44.3	338	36.3	
Colostrum after birth		*n* = 921		*n* = 929		
	Yes	857	93.1	901	97	< 0.001
	No	64	6.9	28	3	
Pre lacteal feeds such as honey, sugar water after birth		*n* = 922		*n* = 924		
	Yes	116	12.6	120	13	0.794
	No	806	87.4	804	87	
**Nutritional status of children**						
Wasting		*n* = 915		*n* = 905		
	Normal	808	88.3	736	81.3	< 0.001
	Wasted	107	11.7	169	18.7	
Stunting		*n* = 914		*n* = 911		
	Not Stunted	561	61.4	722	79.3	< 0.001
	Stunted	353	38.6	189	20.7	
Underweight		*n* = 919		*n* = 916		
	Normal	668	72.7	691	75.4	0.179
	Underweight	251	27.3	225	24.6	
**Morbidity history of the child**						
Diarrhoea in the last 15 days of survey		*n* = 923		*n* = 931		
	Yes	104	11.3	94	10.1	0.414
	No	819	88.7	837	89.9	
Fever in the last 15 days of survey		*n* = 923		*n* = 931		
	Yes	234	25.4	169	18.2	< 0.001
	No	689	74.6	762	81.8	
Cough in the last 15 days of survey?		*n* = 923		*n* = 931		
	Yes	373	40.4	188	20.2	< 0.001
	No	550	59.6	743	79.8	
**Childcare practices during illness and others**						
Oral Rehydration Solution (ORS) given in case of diarrhoea		*n* = 890		*n* = 915		
	Yes	346	38.9	392	42.8	0.087
	No	544	61.1	523	57.2	
Hand washing Practices after cleaning Child defecation		*n* = 917		*n* = 930		
	With soap	371	40.5	475	51.1	< 0.001
	With soil or ash	28	3.1	25	2.7	
	Only with water	518	56.5	430	46.2	
Hand washing practices before feeding the child		*n* = 894		*n* = 349		
	With soap	299	33.4	162	46.4	< 0.001
	With soil or ash	12	1.3	7	2	
	Only with water	574	64.2	168	48.1	
	Don’t wash	9	1	12	3.4	

*Chi square test was used **P < 0.05 considered statistically significant

### Comparison of maternal and child outcomes between the two groups through logistic regression analysis

Mothers in ALS group (AOR 1.71; 95% CI 1.21–2.43) were at increased odds of having more than eight ANC visits compared to mothers in JSY group. Mothers in ALS group were at decreased odds of having complications like severe pain in abdomen (AOR 0.43; 95% CI 0.22–0.86), swelling of legs or feet (AOR 0.59; 95% CI 0.44–0.80) compared to mothers in JSY group. Mothers in ALS group had a decreased odds (AOR 0.004; 95% CI 0.001–0.013) of delivering at a private hospital compared to mothers in JSY group. Children of mothers in ALS group had increased odds of receiving breast-feeding within 30 min of the birth (AOR 1.46; 95% CI 1.13–1.88), receiving colostrum after birth (AOR 2.05; 95% CI 1.18–3.56) compared to children of mothers in JSY group (Table 4).

**Table 4:** Comparison of maternal, neonatal, and infant outcomes between the beneficiaries of Janani Suraksha Yojana and Arogya Laxmi scheme in urban settlements of Hyderabad through univariate and multivariable logistic regression analysis

Variable	Category	Unadjusted odds ratio (95%CI)	Adjusted Odds ratio (95% CI)
Religion	Hindu	Ref.	Ref.
	Muslim	0.89 (0.68–1.15)	1.13 (0.78–1.66)
	Christian/Others	0.44 (0.23–0.70)	0.65 (0.37–1.14)
Category	SC	3.38 (2.18–5.26)*	3.07 (1.76–5.35)*
	ST	4.98 (3.12–7.93)*	5.07 (2.78–9.23)*
	OBC	4.32 (2.83–5.94)*	3.85 (2.29–6.48)*
	General/others	Ref.	Ref.
Wealth Index	Highest	Ref.	Ref.
	High	0.88 (0.66–1. 18)	0.75 (0.51–1.11)
	Middle	0.99 (0.75–1.30)	0.72 (0.5–1.03)
	Low	0.87 (0.67–1.12) *	0.63 (0.45–0.88) *
	Lowest	0.79 (0.58–1.06) *	0.608 (0.41–0.899)*
Total number of ANC visits	≤ 8	Ref.	Ref.
	> 8	2.48 (1.90–3.25) *	1.71 (1.21–2.43) *
High blood pressure during pregnancy	Yes	0.73 (0.50–1.06)	0.98 (0.6–1.62)
	No	Ref.	Ref.
Severe pain in the abdomen	Yes	0.3 (0.18–0.50) *	0.43 (0.22–0.86) *
	No	Ref.	Ref.
Breathlessness during pregnancy	Yes	0.78 (0.52–1.18)	1.35 (0.75–2.42)
	No	Ref.	Ref.
Swelling of legs or feet during pregnancy	Yes	0.57 (0.45–0.71) *	0.59 (0.44–0.8) *
	No	Ref.	Ref.
Place of delivery	Home	0.09 (0.04–0.22) *	0.1 (0.04–0.28) *
	Government hospital	Ref.	Ref.
	Private Hospital	0.01 (0.001–0.014) *	0.004(0.001–0.013) *
Type of delivery	Normal	Ref.	Ref.
	Caesarean	0.74 (0.62–0.89)	1.15 (0.89–1.48)
Birth Weight	≤ 2500	1.16 (0.87–1.55)	1.06 (0.72–1.53)
	> 2500	Ref.	Ref.
Breast feeding within 30 min of birth	Yes	1.39 (1.16–1.68) *	1.46 (1.13–1.88) *
	No	Ref.	Ref.
Colostrum after birth	Yes	2.4 (1.53–3.78) *	2.05 (1.18–3.56) *
	No	Ref.	Ref.
Pre lacteal feeds such as honey, sugar water after birth	Yes	1.04 (0.79–1.36) *	1.68 (1.13–2.49) *
	No	Ref.	Ref.
Stunting	Not Stunted	Ref.	Ref.
	Stunting	0.42 (0.34–0.51) *	0.36 (0.28–0.47) *

*P < 0.05 considered statistically significant

## Discussion

The study has shown that the newly launched ALS scheme had a significant effect on improving antenatal care and increasing institutional births in government facilities compared to the existing JSY scheme. Pregnant women availing ALS scheme had lower pregnancy related complications compared to JSY scheme. Children born to mothers utilizing ALS scheme had better breastfeeding practices and decreased morbidity compared to the children born to mothers utilizing only JSY scheme.

We observed that higher number of mothers in the ALS group had ANC visits at government health facilities (98.6%) compared to mothers in the JSY group (71.5%). The study found that the scheme improved adherence to certain sets of conditions, especially during pregnancy similar to the findings of Kalyani Raghunathan et al. in a study conducted in the state of Odisha [23]. It also increased registration of pregnancies, utilization of antenatal services, and receipt of IFA supplements from government health workers. It is encouraging to know that cash-transfer schemes such as ALS increased the utilization of ANC services, as ANC is a health intervention that is not considered to be equitably distributed in low and middle income countries [24].

It was further observed that only 55.9% of the deliveries of the JSY group happened in a government hospital compared to 99% in the ALS group. We hypothesize that this was because of the better service-utilization incentives. This also indicates that the scheme is reaching the target population which earlier preferred deliveries in private hospital due to lack of awareness. In these groups of population that are less likely to use maternal and child health services, we have demonstrated the role of cash transfer programs like ALS. Another important issue in India with respect to CCT is the quality of care in the health facilities. The existing JSY program improved institutional deliveries, but positive effects were not so significant on maternal mortality. Critics believe this is the result of poor quality of care that pregnant women obtain at government health facilities during delivery [25]. The most common complication among pregnant women was swelling of legs, which was seen more in the JSY group compared to ALS. The increased likelihood of mothers in ALS group receiving ANC services might have led to this reduction in complications during pregnancy.

In our study, the Arogya Laxmi scheme (ALS) availed group had significantly lower Low Birth Weight (LBW) babies compared to JSY group. A significant increase in ANC visits among the women of ALS group might have played a key role in reducing the Low-Birth-Weight babies. These findings are similar to the findings in the study conducted by Paula von Haarenthe in the districts of India [26]. Initiating of breastfeeding within the first half an hour after birth was higher in ALS group compared to the JSY group. Further, a higher proportion in the ALS group fed colostrum to the new-born compared to the JSY group. Antenatal counselling received by the mothers from ALS group contributed the positive effects of early initiation and exclusive breastfeeding to the child born to mother from ALS group.

Child Nutrition and health were also better in the ALS group. More children in the JSY group had fever, diarrhea, and ARI than in the ALS group. Hospitalization of the child due to illness was more commonly recorded in the JSY group than in the ALS group. More mothers in the ALS group consulted government doctors compared to the JSY group. There was also increased awareness of giving ORS in the ALS group compared to the JSY group. Service-utilization incentives are a proactive approach by the government to enable the beneficiaries to use the services which is in opposite to the private clinics where service delivery happens only on payment. Our findings mean that the positive effects of the scheme were not only seen in the increased usage of maternity and immunization services, but also acted as a reinforcement in encouraging people towards more care-seeking behaviors and away from homecare. More hospital visits can also lead to increased health related knowledge, attitude, and practice. These findings in the study of increased health seeking behavior in mothers of ALS group are similar to findings in the study conducted in Tamil Nadu by Rajan Srinivasan et al. [27].

While this approach has improved ANC visits, efforts can be made to provide incentives on the knowledge of the mother on key IYCF indicators. Increased ANC visits decrease morbidity and mortality and improve knowledge on various important nutrition health issues discussed during counseling, which could be transferred to other members of the society. The increased early initiation of breast-feeding improves neonatal morbidity and mortality and can create healthier adults. Reduced expenditure for pregnancy can be used for better nutrition and health of the mother and child or other members of the family. All these factors have an impact on improving the overall community health.

To the best of our knowledge, our study results are the first quantitative estimates of the impact of the augmented ALS scheme. Because of the geographical and demographic differences between Indian states, the study lacks generalizability as the study was done in an urban setup and may be different in a rural setting. However, the sites chosen were slums which reflect the most neglected population in the urban areas. Other limitations of the study in interpreting healthcare utilization patterns are the chances of recall bias and under-reporting. We could not study the relationship of healthcare utilization to the unmeasured variables such as health system supply-based factors or quality. The long-term effect of ALS Scheme on maternal and child health and nutrition outcomes is unknown due to the initial stages of implementation. There is a need for follow-up studies on program sustainability over a long period of time, including both rural and urban communities. As it involves financial commitments by the government, successive governments need to ensure that programs are continued with quality, intensity, continuity, and coverage. While the study has addressed morbidity and other determinants of maternal and child mortality, the sample size was not sufficient to see the differences in maternal-child mortality which require large-scale studies.

The study shows the importance of Conditional Cash Transfer Schemes. It also shows that the utilization of the services increased with the increase in the monetary benefit to the mothers. Therefore, similar CCT schemes have the potential to reap the same benefits if scaled up to other states and possibly to the global community, though the region-wide differences need to be studied extensively that might affect the outcomes.

## Conclusions

Our study findings suggest that augmented Arogya Laxmi Scheme led to increased use of the government health care facilities among pregnant women. ALS has also decreased maternal and neonatal complications during antenatal and postnatal period compared to JSY. In this study, there seemed clear benefits for mothers and their children availing the Augmented ALS scheme compared to the JSY scheme. Longitudinal studies across the state including both rural and urban communities are required to study the impact of Arogya Laxmi Scheme in improving maternal and child health in the long term.

## Data Availability

Data sets used for the analysis of the study is available upon reasonable request to the corresponding author (geddambabuj@gmail.com).

## Abbreviations

ALS: Arogya Laxmi Scheme
ANC: antenatal care
AWC: Anganwadi Centre
CCT: Conditional Cash Transfer
IFA: Iron and Folic Acid
IMR: Infant Mortality Rate
JSY: Janani Suraksha Yojana
LBW: Low Birth Weight
LPG: Liquid Petroleum Gas
MCP: Mother and Child Protection (MCP)
NMR: Neonatal Mortality Rate
OBC: Other Backward Class
OOPE: Out of Pocket Expenditure
ORS: Oral Rehydration Solution
PHC: Primary Health Centre
SC: Schedule Caste
ST: Schedule Tribe
TT: Tetanus Toxoid
